# Cortical and Subcortical Alterations in Medication Overuse Headache

**DOI:** 10.3389/fneur.2018.00499

**Published:** 2018-06-25

**Authors:** Jan Mehnert, Julia Hebestreit, Arne May

**Affiliations:** Department of Systems Neuroscience, University Medical Center Eppendorf, Hamburg, Germany

**Keywords:** analgesic-overuse headache, orbitofrontal cortex, ventromedial prefrontal cortex, nociception, gray matter volume, functional connectivity

## Abstract

Medication-overuse headache is an increasing problem in headache clinics and therapy includes drug withdrawal. Although it has been shown that the orbitofrontal cortex is hypo-metabolic and exhibits less gray matter in these patients the functional role of this finding is still unclear as virtually no functional imaging studies exploring withdrawal of medication have been published. We compared structural and functional magnetic resonance images of 18 patients before and after drug withdrawal with age and gender matched controls using a well-established trigeminal, nociceptive fMRI paradigm. We reproduced structural changes in the orbitofrontal cortex of the patients which highly correlated with the clinical outcome of medication withdrawal. The neuronal activity before drug withdrawal in pain related regions (operculum, insula, spinal trigeminal nucleus) was reduced compared to after drug withdrawal and the orbitofrontal cortex showed a reduced functional connectivity to the nociceptive input region (spinal trigeminal nucleus) and the cerebellum which regained after withdrawal. These data suggest the seminal role of the orbitofrontal cortex as a mediator between bottom-up and top-down stream in headache processing.

## Introduction

Migraine patients who use analgesics and/or triptans on a regular basis are at risk to develop a medication overuse headache (MOH) (1). Medication overuse facilitates the transition from episodic to chronic migraine (2, 3), which is reversible if medication is withdrawn (4). Neuronal mechanisms underlying MOH may help understanding this transition. Early results using glucose positron emission tomography (PET) imaging showed hypo-metabolic orbitofrontal cortex (OFC) activity before but also following withdrawal (5), which has been interpreted as a risk factor for ongoing medication overuse and possibly relapse of MOH. That this finding is indeed rather specific for MOH patients is underpinned by further findings using a different imaging method, namely structural magnetic resonance imaging (MR), which showed decreased gray matter volume in the same region (6–8). Recent studies on resting-state functional connectivity alterations in MOH patients further suggest an altered connectivity of the OFC with the precuneus (6), nucleus accumbens (9), and parahippocampal gyrus (10). However, virtually no functional imaging data of MOH patients exists, with the possible exception of one cohort study which compared MOH patients with healthy controls but did not directly compare before and after withdrawal in patients (11).

We therefore focused on the question of possible changes in central trigeminal pain processing in MOH patients before and after medication withdrawal.

## Materials and methods

### Subjects and experimental design

Eighteen patients suffering from medication overuse headache and migraine (with and without aura) were recruited in the headache outpatient department of the University Clinic of Hamburg Eppendorf and scanned at two time points: before and after medication withdrawal. The time points of scanning had to differ for a minimum duration of 8 weeks to allow the efficacy of drug withdrawal. In the patient group, we assessed the number of headache days in the month before the first scan and continuously until the second scan. The number of headache days in the month before each scan were taken as measure of withdrawal success. We also assessed which and how often analgesia were used and whether and when a preventative medication was started. Two patients already used preventative medication (1 Flunarizin, 1 Venlaflaxin in combination with Valproic Acid) at the first measurement, while 11 patients used preventative drugs at the second time point after drug withdrawal (4 Metropolol, 4 Flunarizin, 1 Amitriptylin, 1 Amitriptylin in combination with Flunarizin, and 1 Venlaflaxin in combination with Valproic acid) leaving 7 without preventative medication. Patients further completed German versions of the Becks Depression Inventory (DBS-II) (12) and a symptom checklist (SCL-90-R) (13) to assess changes in depression and anxiety scores. Anxiety was measured as the average score in 10 anxiety related questions (questions: 2, 17, 23, 33, 39, 57, 72, 78, 80, 86). Additionally, 18 age- and gender matched healthy controls without a history of headache were invited to participate and were also scanned twice at the same interval as the patients to control for time and session effects. Controls were free of any neurological or psychiatric diseases, did not suffer from headache and had no 1st grade relatives suffering from primary headache. Details regarding clinical data can be found in Table 1.

**Table 1:** Clinical characteristics of participants.

**Number of participants (sex)**	**Patients**	**Controls**
	**18 (13 female, 5 male)**	**18 (13 female, 5 male)**
Age (mean ± SD)	36 ± 14	34 ± 9
Duration (days) between first and second time point (mean ± SD)	108 ± 101	87 ± 38
With/without aura	4/14	
Years suffering from migraine (mean ± SD [range])	20 ± 14 [0, 51]	
Acute medication before withdrawal (triptans/NSAIDs/both)	5/12/1	
Days per month using acute medication at 1st/2nd scan (mean ± SD [range])	17 ± 6 [11,30]/5 ± 3 [0, 10]	
Days per month with headache before withdrawal (mean ± SD (range])	21 ± 4 [14,30]	
Days per month with headache after withdrawal (mean ± SD [range])	10 ± 5 [1,17]	
Reduction of headache days per month (mean ± SD [range])	12 ± 5 [4, 24]	
Relative reduction of headache days per month (mean percentage ± SD [range])	54 ± 20 [19, 96]	
Use of preventative medication at 1st/2nd scan	2/11	
Use of acute medication at first/second time point (within 24 h)	7/1	
Headache at day of scanning at first/second time point (within 24 h)	9/2	
Days before last attack at 1st/2nd scan (mean ± SD [range])	3 ± 6 [0, 26]/11 ± 17 [0, 70]	
Days until next attack at 1st/2nd scan (mean ± SD [range])	8 ± 11 [1, 43]/7 ± 6 [0, 23]^a^	
Becks depression inventory at 1st/2nd scan (mean ± SD [range])	9 ± 7 [1, 25]/7 ± 6 [0, 23]^b^	
Anxiety score of SCL-90-R at 1st/2nd scan (mean ± SD)	0.38 ± 0.42/0.30 ± 0.28^c^	

SD, standard deviation; NSAID, non-steroidal anti-inflammatory drugs.

a *data not available for 6 patients*.

b *data not available for 1 patients*.

c *data not available for 2 patients*.

The experiment followed a well-established paradigm inducing trigeminal nociception which has been published before in detail (14–17). In short, volunteers were exposed to 15 standardized trigeminal nociceptive stimuli (gaseous ammonia) and several control stimuli through an air-proofed Teflon tube which was, on one end, connected to an olfactometer outside of the scanning room. Control conditions were either an olfactory (rose odor), an odorless (air puffs) stimulation (both used 15 times for 1 s each), or a visual stimulus (flickering checkerboard, shown 16 times for 4 s each). All conditions were pseudo-randomly presented with a jittered time lag and after each stimulus the participants rated the stimulus for intensity and unpleasantness on a visual analog scales reaching from 0 to 100. As we focused on nociceptive processing, we only analyzed ratings of the painful stimulations and used two-tailed two sample *t*-test for both time points individually to assess differences between healthy volunteers and patients at these two time points. The protocol was approved by the Ärztekammer Hamburg (PV2814). All subjects gave written informed consent in accordance with the Declaration of Helsinki.

### MR data acquisition

Both sessions were identical. All subject were instructed how to perform the experiment and went into the 3 Tesla MR Scanner (TIM TRIO, Siemens, Germany). Here, high resolution MPRAGE (1 mm^3^) structural images and functional images (echo planar imaging sequence, repetition time 2.62 s, echo time 30 ms, flip angle 80°, field of view 220 × 220 mm^2^, 2 × 2 mm^2^ in-plane resolution, 40 axial slices per volume with 2 mm slice thickness, and 1 mm gap) during the actual experiment were acquired in approximately 55 min. Further details on the MR parameters can be found elsewhere (18–20).

### Voxel based morphometry analysis

Structural scans from both sessions were analyzed with the Computational Anatomy Toolbox (CAT12 toolbox by Christian Gaser, http://www.neuro.uni-jena.de/cat/) for statistical parametric mapping (SPM12, http://www.fil.ion.ucl.ac.uk/spm/software/spm12/) to assess gray matter volume alterations by means of voxel based morphometry (VBM) (21, 22). For this approach the structural image from both sessions of each subject were co-registered to their mean, segmented and normalized to MNI space and modulated to preserve gray matter volume. Resulting images had an isotropic resolution of 1.5 mm^3^ and were then smoothed by an 8 mm^3^ isotropic Gaussian kernel and entered the statistical group comparison. Total intracranial volume was extracted from the CAT12 results and used as a covariate of no interest for all following statistical analyses. Statistical analyses included (1) two sample *t*-tests for the first as well as second session to compare the group means, flexible factorial analysis of variance (ANOVA) with the factors subject, group and time to statistically assess (2) differences between time points for both groups individually as well as (3) the interaction of the factors group and time (group × time). Results are presented at a statistical threshold of *p* < 0.05 family wise error (FWE) small volume corrected (using a sphere with 12 mm radius as search volume and an initial voxel-level threshold of *p* < 0.001) with a cluster extent of more than 30 voxel (101.25 mm^3^ ~ 0.10 mL). Region of interest were hypothesized from the current available literature on medical overuse headache and used as coordinates for the small volume correction.

Regions of interest resulting from the VBM analysis were further correlated with the reduction of headache days per month (absolute and percentage) by means of Spearman's correlations to assess their predictability of the clinical outcome.

### Functional MR data processing and analyses

The functional images of both sessions were denoised, using spatially adaptive non-local means (23) denoising (implemented in the aforementioned CAT12 toolbox), realigned and slice time corrected. The structural image from the first session was co-registered to the mean image of both session, segmented and normalized to MNI space. Normalization parameters of the structural images were applied to all functional images, warping also them to MNI space. Afterwards functional images had an isotropic resolution of 2.0 mm^3^ and were smoothed with an 8 mm^3^ isotropic Gaussian kernel and entered a general linear model (GLM) analysis. Here 5 conditions (ammonia, air puffs, rose odor, visual stimulation, and all button presses) were modeled by convolving a hemodynamic response function (implemented in SPM12) with the onsets of the stimuli and fitting them to the actual data.

Contrast images from the condition pain and air puffs (differences of their beta-images which result as fitting parameters from the GLM analysis) signifying strength of neuronal activity during nociception entered the statistical group comparison, modeled similar to the one in the VBM analyses, including (1) two sample *t*-tests for the first as well as second session to compare the group means, flexible factorial ANOVA with the factors subject, group and time to statistically assess (2) differences between time points for both groups individually as well as (3) the interaction of the factors group and time (group × time). As for the VBM analyses, results for the functional imaging are presented at a statistical threshold of *p* < 0.05 FWE small volume corrected (using a sphere with 12 mm radius as search volume and an initial voxel-level threshold of *p* < 0.001) with a minimum cluster extent 30 voxel (240 mm^3^ $\overset{\wedge }{=}$ 0.24 mL). Region of interest were hypothesized from the current available literature on medical overuse headache and used as coordinates for the small volume correction.

Alterations of functional connectivity of the OFC were analyzed using Psychophysiological Interaction (PPI) analysis as implemented in SPM12 (24) and statistically tested using aforementioned ANOVA. Here we used the coordinates from the original work of Fumal (5) and colleagues with a sphere of 12 mm radius as seed region and the contrast between the nociceptive (ammonia) and control (air puffs) condition as psychological variable. The same aforementioned statistical thresholds were used. The coordinates for the seed of the PPI was not taken from the VBM results of this data-set to prevent circular reasoning (25).

To investigate coherences between structural and functional features we calculated an exploratory regression analysis using baseline GMV of the OFC cluster as a covariate for the contrast nociception at time point 1 > time point 2.

## Results

### Behavioral results

Age and time interval between scans of patients and controls were not significantly different at an alpha level of α = 0.05. As we were specifically interested in withdrawal, we only included patients into this analysis who were successful in withdrawing medication. Patients who were unsuccessful in medication withdrawal (*n* = 2 out of 20 patients) withdrew consent to be scanned a second time and were not available for statistical comparison. Between scans, the patients showed a reduced headache frequency after withdrawal of 12 ± 5 days per month on average which equaled 54 ± 20 % (mean ± standard deviation) relative headache reduction (RD) calculated as RD = (T1–T2)/T1^*^100 where T1 signifies the number of headache days per month at time point 1 and T2 at time point 2, respectively (Table 1). The reduction corresponds to a change from a chronic to an episodic migraine. Pain ratings were significantly higher in the patient than in the control group only at the first time point (*p* = 0.029 for intensity and *p* = 0.047 for unpleasantness, respectively) at an alpha level of α = 0.05. However, there were no differences within the groups between the first and second time point. Pain intensity as well as unpleasantness ratings were available in all but one patient. Neither anxiety (SCL-90-R) nor depression (DBS-II) scores were significantly different between the two scanning times for the patient group.

### Gray matter volume alterations

**Figure 1:**
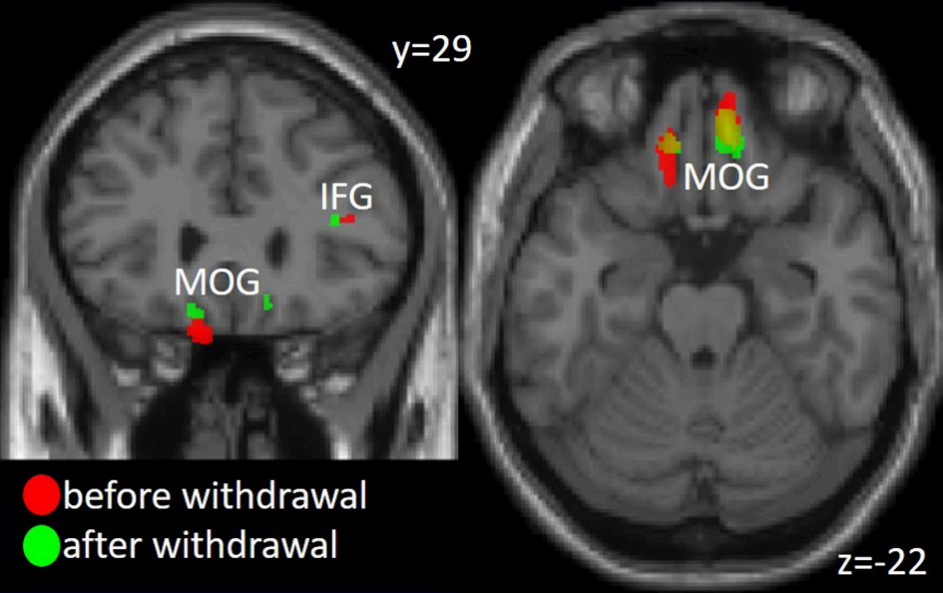
Gray matter volume decrease in Medial Orbital Gyrus (MOG) and Inferior Frontal Gyrus (IFG) in the patient group compared to the healthy controls before and after medication withdrawal. The threshold for visualization is set to *p* < 0.001 uncorrected with a minimal cluster extent of 20 voxel.

Decreased gray matter volume in patients as compared to controls in the first time point (before drug withdrawal) were found in the hippocampus, precuneus, inferior frontal gyrus (IFG), and bilateral orbitofrontal cortex (OFC), more specifically the medial orbital gyrus (MOG). The comparison between patients and controls at time point 2 (after a minimum of 8 weeks of drug withdrawal) revealed similar results namely decreased gray matter volume in OFC, hippocampus, IFG and additionally superior frontal and middle temporal gyrus and the cerebellum. However, an interaction analysis between the factors group and time did not reach significance. See Figure 1 and Table 2 for details.

**Table 2:** Gray matter volume (GMV) alterations of MOH patients by means of voxel based morphometry (VBM) analysis.

**Cluster size (voxel with 1.5 mm^3^)**	***T*-value**	**MNI coordinate**			**Region (reference used for small volume correction)**
		**x**	**y**	**z**	
**GMV decrease in patients before drug withdrawal (time point 1) compared to controls**					
287	4.42	30	−21	−9	r hippocampus (6)
344	4.37	−12	38	−24	l medial orbital g (5)
294	4.25	10	48	−22	r medial orbital g (5)
35	4.04	14	−54	51	r precuneus (8)
64	3.98	40	32	12	r inferior frontal g (6)
31	3.85	−20	−15	−9	l hippocampus (6)
**GMV decrease in patients after drug withdrawal (time point 2) compared to controls**					
271	4.61	10	45	−21	r medial orbital g (5)
319	4.61	12	−92	−15	r lingual g (26)
44	4.29	−50	−68	−45	l cerebellum (lobule VIIIa) (14)
204	4.22	28	−18	−12	r hippocampus (6)
209	4.17	−12	38	−20	l medial orbital g (5)
62	3.94	38	30	14	r inferior frontal g (6)
49	3.90	8	14	72	r superior frontal g (6)
**GMV decrease in patients during drug withdrawal (after**<**before)**					
170	4.54	0	−70	18	l cuneus (26)
47	4.44	−48	−30	3	l superior temporal g (26)
72	4.06	−36	−48	−51	l cerebellum (lobule VIIIa) (14)
**GMV increase in patients during drug withdrawal (after**>**before)**					
*n.s*.					
**GMV alterations in healthy controls**					
*n.s*.					

*l, left; r, right; g, gyrus; n.s., not significant*.

The comparison of gray matter volume before and after drug withdrawal revealed an *additional* decrease of gray matter volume in 4 clusters including cuneus, superior temporal gyrus, putamen, and cerebellum (Table 2). The healthy participants showed no significant changes in gray matter between the first and second time point.

### Predictability of clinical outcome

**Figure 2:**
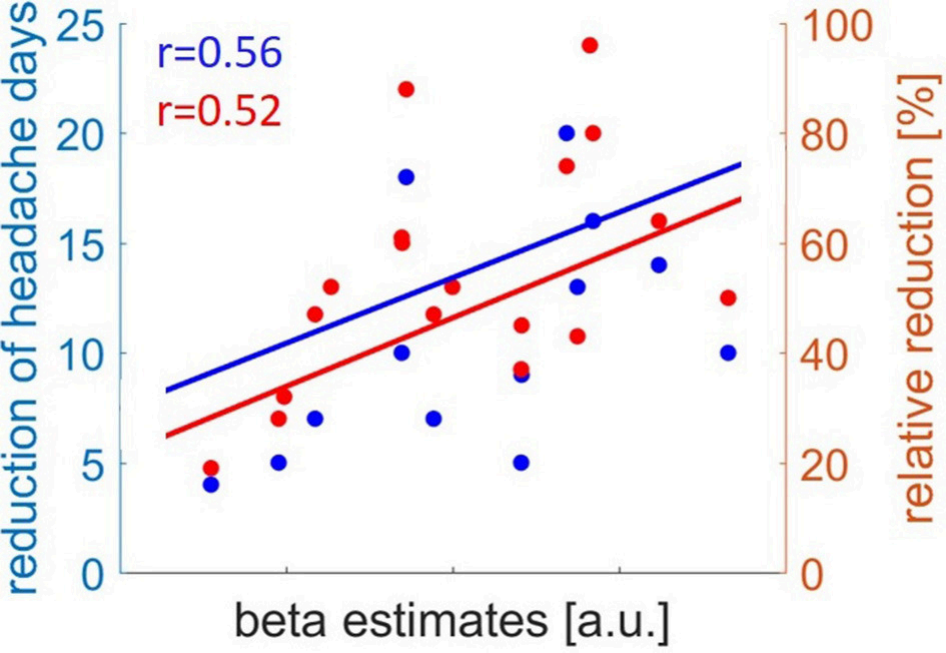
Correlation of gray matter volume in Medial Orbital Gyrus before drug withdrawal and clinical outcome of headache reduction. Absolute reduction of headache days in blue and relative reduction of headache days in red.

The gray matter volume of the right MOG before withdrawal showed a positive relation with the clinical outcome in reduction of headache days per month (absolute) (*r* = 0.560, *p* = 0.016) and relative reduction (RD)(*r* = 0.515, *p* = 0.029) (Figure 2).

### Neuronal activity in nociceptive processing

**Figure 3:**
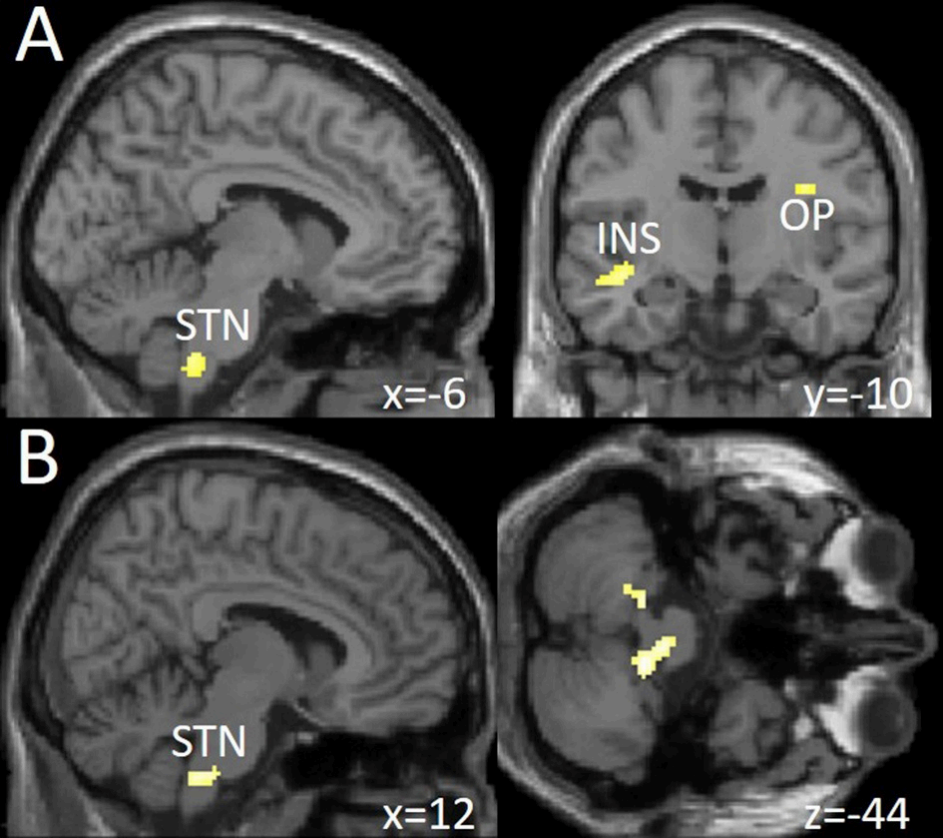
**(A)** Increased neuronal activity following trigeminal nociceptive input after drug withdrawal in spinal trigeminal nucleus (STN), insula (INS), and operculum (OP) at a visualization threshold of *p* < 0.001 uncorrected. **(B)** Increase of functional connectivity by means of a psychophysiological interaction (PPI) analysis from OFC/MOG to spinal trigeminal nucleus (STN) and the cerebellum during drug withdrawal in the patient group. Shown at a visualization threshold of *p* < 0.001, uncorrected. Coordinates are given according to the MNI system.

We found a significantly increased activation in the left spinal trigeminal nucleus (sTN), right operculum (lowering threshold bilateral), and left posterior insula after withdrawal compared to before (Figure 3A and Table 3). No change of BOLD signal was found in the control group between time points. The Interaction analysis of nociceptive stimulation of group x time showed no significant effect at the chosen threshold similar to the comparison between healthy controls and patients at both time points.

**Table 3:** Alteration in neuronal responses to nociception and in functional connectivity from OFC in MOH patients and controls.

**Cluster size (voxel with 2 mm^3^)**	***T*-value**	**MNI coordinate**			**Region (reference used for small volume correction)**
		**x**	**y**	**z**	
**Neuronal activity in patients (after**>**before drug withdrawal)**					
54	4.52	36	−6	24	r operculum (14)
77	4.28	−42	−12	−10	l SII (17)
142	4.05	−4	−38	−48	l spinal trigeminal nucleus (16)
**Neuronal activity in patients (after**<**before drug withdrawal)**					
*n.s*.					
**Neuronal activity in patients vs. controls at both time points**					
*n.s*.					
**Interaction in neuronal activity in: group** × **time**					
*n.s*.					
**Functional connectivity from OFC in patients (after** >**before drug withdrawal)**					
132	4.17	12	−38	−44	r spinal trigeminal nucleus and cerebellum (16)
30	3.83	−14	−38	−44	l cerebellum (14)

*l, left; r, right; b, bilateral; n.s., not significant*.

### Changes in functional connectivity during nociception

Following medication withdrawal, e.g., at time point 2 the right MOG showed a significantly increased connectivity to the right STN, bilateral cerebellum in the patient group only. See Table 3 and Figure 3B.

### Coherence

Taking the average value of the baseline GMV of the individuals' left OFC cluster (coordinates −12, 38, −24) as a covariate for the contrast (ammonia-air)_post_-(ammonia-air)_pre_ did not show any significant results in the F-contrast using a voxel-wise FWE-corrected threshold of *p* < 0.05. Lowering the threshold to an uncorrected threshold of *p* < 0.001 and a minimum cluster size of 30 voxel in the negative T-contrast (i.e., lower GMV of the OFC at baseline explains variance of increase in nociceptive activation) reveals 6 clusters, namely: left planum polare, left anterior insula, right cerebellum and STN, right operculum, right fusiformis gyrus, and left posterior insula. Patients and controls were analyzed together. Supplementary Image 1 shows these results superimposed on an MRI).

## Discussion

**Figure 4:**
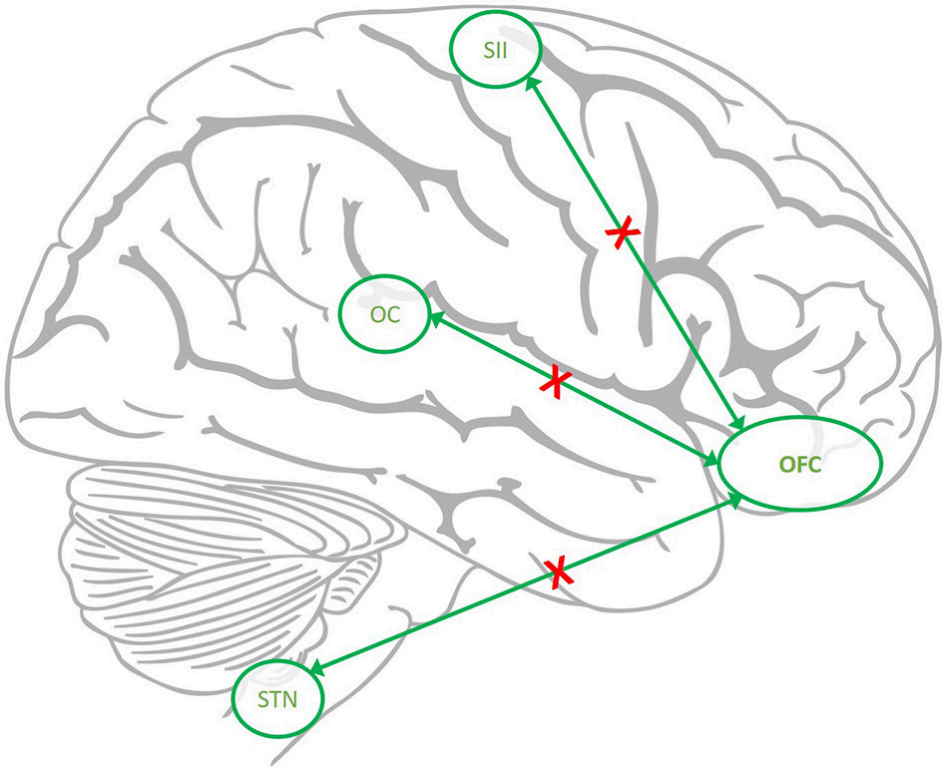
The hypothesized down regulated network in MOH. The connectivity from orbitofrontal cortex (OFC) to spinal trigeminal nucleus (STN), Operculum (OC), and somatosensory cortex (SII) is reduced (red crosses) in MOH but recovers after withdrawal.

Our main finding is a better characterization of the functional consequences of earlier findings of hypo-metabolism of the orbitofrontal gyrus in patients with medication overuse headache which persist after medication withdrawal (5). We could reproduce earlier findings of a decrease of gray matter volume in this region (7, 8) and could reproduce that the extent of volume loss in this region correlates to the outcome after withdrawal (7). The hypo-metabolism and structural gray matter loss in the same brain area strongly suggests that a dysfunction of this area is indeed specific for MOH as it has not been reported in primary headache disorders (27). We therefore focused on possible functional imaging changes in MOH patients before and after withdrawal and contrasted these findings with findings of healthy controls who underwent an identical scanning protocol. Following trigeminal nociceptive input patients showed an increased neuronal activity in the spinal trigeminal nucleus, posterior insula (equaling somatosensory cortex, SII) and operculum in the episodic state, after drug withdrawal compared to the first scan. This could be due to the fact that significantly more patients suffered from acute headache on the first scan (*n* = 9) than on the second scan (*n* = 2). However, the behavioral data, i.e., the pain ratings did not change significantly between the first and the second scan. Interestingly, the increase in activity in the spinal trigeminal nucleus, SII and operculum was accompanied by a slight increase of activity in the orbitofrontal cortex, which however did not reach significance level. Given that analgesics as such do not change the neuronal pattern of these trigeminal pain transmitting areas even using the same experimental protocol (28, 29), we speculate that the robust metabolic and structural changes in the orbitofrontal cortex form a functional network together with the spinal trigeminal nucleus, somatosensory cortex and operculum and that these changes reflect subdued activity under regular intake of pain medications as sketched in Figure 4. One could argue that patients suffered more headache days per months before withdrawal but if nociceptive transmission due to headache would have changed such a functional network one would have assumed the opposite- more activity at scan 1–in this network.

Given that the orbitofrontal cortex plays such a robust role in MOH patients, we additionally performed a PPI analysis. Contrary to the main analysis; the PPI analysis focuses on the functional connectivity between the seed region (here: orbitofrontal cortex) and other pain related brain areas during pain. In the present study we show a down regulatory mechanism of the functional connectivity between the orbitofrontal cortex and the trigeminal nociceptive brainstem nuclei and opercular and secondary somatosensory cortex. It seems that regular intake of pain killers initiates a central regulatory mechanism resulting in an attenuated coupling i.e., functional connectivity between the OFC and brain regions known to be altered in migraineurs (30). Importantly, this alteration reversed following medication withdrawal, e.g., at time point 2 the right MOG showed a significantly increased connectivity to the STN, and bilateral cerebellum in the patient group only.

Several recent imaging studies suggested an altered coupling between brain areas in MOH patients (6, 9, 10). However, these studies used several different methods (for example resting state) investigating functional connectivity between cortical regions and are therefore not directly comparable to each other and our own results. We specifically looked for an increased coupling of the OFC in both conditions: MOH and after withdrawal and found a significant change only after withdrawal. One could speculate that this specific inhibition of OFC-trigemino-cortical functional coupling is due to the regular intake of pain killers and acts on a brain network that computes sensory modalities (31). If this is a general phenomenon, MOH patients should be more pain sensitive also in areas outside of the trigeminal territory. Our finding of a decreased coupling of the trigeminal nociceptive nucleus with the OFC in MOH patients would then be due to the fact that we tested trigeminal nociceptive and not somatic input. And indeed MOH patients show changes in pressure-pain thresholds and supra-threshold pain scores for electrical stimulation not only in cephalic but also extra-cephalic regions (32). Interestingly, temporal summation (an indicator for sensitization) normalized in MOH patients after detoxification (32).

The fact that drug dependence and addiction also are accompanied by metabolic changes in the orbitofrontal cortex (33) puts analgesic overuse close to addiction (8, 34). Of note, whether the changes seen in functional imaging in chronic migraine and MOH patients are cause or consequence of regular analgesic intake is not clear. Moreover, headache patients take analgesic medication not for emotional or psychedelic effects but to counteract pain which is the reason why clinicians do not talk about addiction in MOH patients. However, the possible common ground could be that the orbitofrontal cortex is connected with brain areas that compute sensory modalities and mediate executive function and can therefore serve to integrate the physical and emotional attributes of a stimulus (33). A dysfunction of this area could result in a dysregulation of the ability to evaluate potential reward against harm from drug self-administration. It is certainly oversimplifying its role in addiction, substance abuse, craving and impaired decision making, but the common ground could well be that the immediate value of pain reduction would be set against the possible long-term result of side effects.

### Limitations

The interpretability of the results of this study needs to take the relatively small number of subjects into consideration, although the longitudinal approach allows taking each patient (and healthy participant) as their own controls and such reducing the variance. A possible limitation is that we investigated patients with and without a preventative medication. Ideally, such a study would only include patients who are medication free. However, as for ethical reasons the imaging study had to follow clinical decisions, some patients were on preventative medication to help decreasing migraine frequency. Given that preventative medications did not show functional changes in the areas we found in MOH patients but rather changes in the thalamus (18) and hypothalamus (19), we do not think it plausible that preventatives alone explain our findings. On the whole there are few imaging studies in MOH. Our data add to these findings that the OFC shows a decreased functional connectivity between the OFC and top-down and bottom-up pain processing regions namely the STN and cerebellum. Future studies should try to investigate patients who do not take preventatives.

## Author contributions

JM: analysis and interpretation of data, drafting of the manuscript; JH: acquisition of data, analysis and interpretation of data, drafting of the manuscript; AM: conception and design of the study, interpretation of data, revising the manuscript critically for important intellectual content.

## Data availability statement

The raw data supporting the conclusions of this manuscript will be made available by the authors, without undue reservation, to any qualified researcher.

### Conflict of interest statement

The authors declare that the research was conducted in the absence of any commercial or financial relationships that could be construed as a potential conflict of interest.

## Supplementary material

Supplementary Image S1 Regression analysis of the covariation between an increase of activation in response to nociception and reduction of baseline GMV in OFC at an uncorrected threshold of *p* < 0.001 and a minimum cluster extent of 30 voxel.

## References

[1] Headache Classification Committee of the International Headache Society (IHS). The international classification of headache disorders, 3rd edition. Cephalalgia (2018) 38:1–211. 10.1177/0333102413485658 29368949

[2] Bigal ME Lipton RB. When migraine progresses: transformed or chronic migraine. Expert Rev Neurother. (2006) 6:297–306. 10.1586/14737175.6.3.297 16533134

[3] May A Schulte LH. Chronic migraine: risk factors, mechanisms and treatment. Nat Rev Neurol. (2016) 12:455–64. 10.1038/nrneurol.2016.93 27389092

[4] Diener HC Limmroth V. Medication-overuse headache: a worldwide problem. Lancet Neurol. (2004) 3:475–83. 10.1016/S1474-4422(04)00824-5 15261608

[5] Fumal A Laureys S Di Clemente L Boly M Bohotin V Vandenheede M. Orbitofrontal cortex involvement in chronic analgesic-overuse headache evolving from episodic migraine. Brain (2006) 129:543–50. 10.1093/brain/awh691 16330505

[6] Chanraud S Scala GD Dilharreguy B Schoenen J Allard M Radat F. Brain functional connectivity and morphology changes in medication-overuse headache: clue for dependence-related processes? Cephalalgia (2014) 34:605–15. 10.1177/0333102413519514 24449748

[7] Riederer F Gantenbein AR Marti M Luechinger R Kollias S Sándor PS. Decrease of gray matter volume in the midbrain is associated with treatment response in medication-overuse headache: possible influence of orbitofrontal cortex. J Neurosci. (2013) 33:15343–9. 10.1523/JNEUROSCI.3804-12.2013 24068801 PMC6618461

[8] Riederer F Marti M Luechinger R Lanzenberger R Meyenburg J von Gantenbein AR. Grey matter changes associated with medication-overuse headache: Correlations with disease related disability and anxiety. World J Biol Psychiatry (2012) 13:517–25. 10.3109/15622975.2012.665175 22746999

[9] Torta DM Costa T Luda E Barisone MG Palmisano P Duca S. Nucleus accumbens functional connectivity discriminates medication-overuse headache. Neuroimage Clin. (2016) 11:686–93. 10.1016/j.nicl.2016.05.007 27330969 PMC4900511

[10] Chen Z Chen X Liu M Dong Z Ma L Yu S. Altered functional connectivity architecture of the brain in medication overuse headache using resting state fMRI. J Headache Pain (2017) 18:25. 10.1186/s10194-017-0735-0 28220377 PMC5318354

[11] Grazzi L Chiapparini L Ferraro S Usai S Andrasik F Mandelli ML. Chronic migraine with medication overuse pre-post withdrawal of symptomatic medication: clinical results and FMRI correlations. Headache (2010) 50:998–1004. 10.1111/j.1526-4610.2010.01695.x 20618816

[12] Kühner C Bürger C Keller F Hautzinger M. Reliabilität und validität des revidierten Beck-Depressionsinventars (BDI-II). Nervenarzt (2007) 78:651–6. 10.1007/s00115-006-2098-7 16832698

[13] Franke GH SCL-90-R. Die Symptom-Checkliste von Derogatis - Deutsche Version [Symptom Checklist-90-R], 2nd Edn. Göttingen: Beltz (2002).

[14] Mehnert J Schulte L Timmann D May A. Activity and connectivity of the cerebellum in trigeminal nociception. Neuroimage (2017) 150:112–8. 10.1016/j.neuroimage.2017.02.023 28192274

[15] Mehnert J May A. Functional and structural alterations in the migraine cerebellum. J Cereb Blood Flow Metab. (2017). [Epub ahead of print]. 10.1177/0271678X17722109 28737061 PMC6446424

[16] Schulte LH Sprenger C May A. Physiological brainstem mechanisms of trigeminal nociception: An fMRI study at 3T. NeuroImage (2016) 124:518–25. 10.1016/j.neuroimage.2015.09.023 26388554

[17] Stankewitz A Voit HL Bingel U Peschke C May A. A new trigemino-nociceptive stimulation model for event-related fMRI. Cephalalgia (2010) 30:475–85. 10.1111/j.1468-2982.2009.01968.x 19673914

[18] Hebestreit JM May A. Topiramate modulates trigeminal pain processing in thalamo-cortical networks in humans after single dose administration. PLoS ONE (2017) 12:e0184406. 10.1371/journal.pone.0184406 28991914 PMC5633146

[19] Hebestreit JM May A. The enigma of site of action of migraine preventives: no effect of metoprolol on trigeminal pain processing in patients and healthy controls. J Headache Pain (2017) 18:116. 10.1186/s10194-017-0827-x 29285569 PMC5745371

[20] Kröger IL May A. Triptan-induced disruption of trigemino-cortical connectivity. Neurology (2015) 84:2124–31. 10.1212/WNL.0000000000001610 25948722

[21] Ashburner J Friston KJ. Voxel-based morphometry—the methods. NeuroImage (2000) 11:805–21. 10.1006/nimg.2000.0582 10860804

[22] Draganski B Gaser C Busch V Schuierer G Bogdahn U May A. Neuroplasticity: changes in grey matter induced by training. Nature (2004) 427:311–2. 10.1038/427311a 14737157

[23] Coupe P Yger P Prima S Hellier P Kervrann C Barillot C. An optimized blockwise nonlocal means denoising filter for 3-D magnetic resonance images. IEEE Trans Med Imaging (2008) 27:425–41. 10.1109/TMI.2007.906087 18390341 PMC2881565

[24] Friston KJ Buechel C Fink GR Morris J Rolls E Dolan RJ. Psychophysiological and modulatory interactions in neuroimaging. NeuroImage (1997) 6:218–29. 10.1006/nimg.1997.0291 9344826

[25] Kriegeskorte N Simmons WK Bellgowan PSF Baker CI. Circular analysis in systems neuroscience: the dangers of double dipping. Nat Neurosci. (2009) 12:535–40. 10.1038/nn.2303 19396166 PMC2841687

[26] Lai TH Chou KH Fuh JL Lee PL Kung YC Lin CP. Gray matter changes related to medication overuse in patients with chronic migraine. Cephalalgia (2016) 36:1324–33. 10.1177/0333102416630593 26853805

[27] May A. New insights into headache: an update on functional and structural imaging findings. Nat Rev Neurol. (2009) 5:199–209. 10.1038/nrneurol.2009.28 19347025

[28] Kröger IL May A. Central effects of acetylsalicylic acid on trigeminal-nociceptive stimuli. J Headache Pain (2014) 15:59. 10.1186/1129-2377-15-59 25201152 PMC4161265

[29] Kröger IL May A. Pharmacological neuroimaging in headache and pain. Curr Opin Neurol. (2013) 26:254–61. 10.1097/WCO.0b013e32836085df 23511443

[30] Schulte LH May A. Functional neuroimaging in migraine: chances and challenges. Headache (2016) 56:1474–81. 10.1111/head.12944 27654831

[31] Hof PR Mufson EJ Morrison JH. Human orbitofrontal cortex: cytoarchitecture and quantitative immunohistochemical parcellation. J Comp Neurol. (1995) 359:48–68. 10.1002/cne.903590105 8557847

[32] Munksgaard SB Bendtsen L Jensen RH. Modulation of central sensitisation by detoxification in MOH: results of a 12-month detoxification study. Cephalalgia (2013) 33:444–53. 10.1177/0333102412475235 23431023

[33] London ED Ernst M Grant S Bonson K Weinstein A. Orbitofrontal cortex and human drug abuse: functional imaging. Cereb Cortex (2000) 10:334–342. 10.1093/cercor/10.3.334 10731228

[34] Calabresi P Cupini LM. Medication-overuse headache: similarities with drug addiction. Trends Pharmacol Sci. (2005) 26:62–8. 10.1016/j.tips.2004.12.008 15681022

