# Supplementary figures and images for: Cortical and Subcortical Alterations in Medication Overuse Headache
Source: Front Neurol. 2018 Jun 25;9:499. doi: 10.3389/fneur.2018.00499 (PMC6026656; doi:10.3389/fneur.2018.00499)

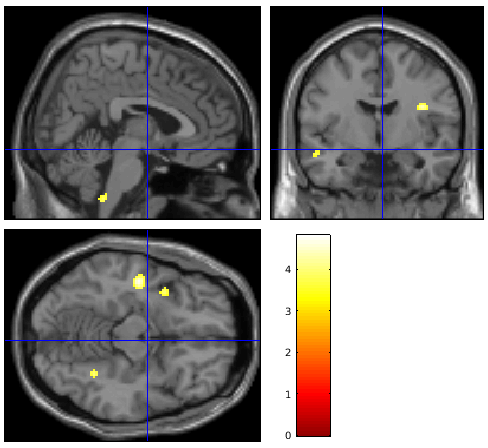

Supplement: Supplementary Image S1 — Regression analysis of the covariation between an increase of activation in response to nociception and reduction of baseline GMV in OFC at an uncorrected threshold of p < 0.001 and a minimum cluster extent of 30 voxel. [file Image_1.PNG]
